# Protective Effects of Black Raspberry (*Rubus occidentalis*) Extract against Hypercholesterolemia and Hepatic Inflammation in Rats Fed High-Fat and High-Choline Diets

**DOI:** 10.3390/nu12082448

**Published:** 2020-08-14

**Authors:** Taehwan Lim, Juhee Ryu, Kiuk Lee, Sun Young Park, Keum Taek Hwang

**Affiliations:** Department of Food and Nutrition, and Research Institute of Human Ecology, Seoul National University, Seoul 08826, Korea; imtae86@snu.ac.kr (T.L.); issue221@snu.ac.kr (J.R.); leku@snu.ac.kr (K.L.); sunyoung.park@snu.ac.kr (S.Y.P.)

**Keywords:** black raspberry, excessive choline, TMAO, hypercholesterolemia, hepatic inflammation

## Abstract

Choline is converted to trimethylamine by gut microbiota and further oxidized to trimethylamine-*N*-oxide (TMAO) by hepatic flavin monooxygenases. Positive correlation between TMAO and chronic diseases has been reported. Polyphenols in black raspberry (BR), especially anthocyanins, possess various biological activities. The objective of this study was to determine the effects of BR extract on the level of choline-derived metabolites, serum lipid profile, and inflammation markers in rats fed high-fat and high-choline diets. Forty female Sprague-Dawley (SD) rats were randomly divided into four groups and fed for 8 weeks as follows: CON (AIN-93G diet), HF (high-fat diet), HFC (HF + 1.5% choline water), and HFCB (HFC + 0.6% BR extract). Serum levels of TMAO, total cholesterol, and low-density lipoprotein (LDL)-cholesterol and cecal trimethylamine (TMA) level were significantly higher in the HFC than in the HFCB. BR extract decreased mRNA expression of pro-inflammatory genes including nuclear factor-κB (NF-κB), interleukin (IL)-1β, IL-6, and cyclooxygenase-2 (COX-2), and protein expression of NF-κB and COX-2 in liver tissue. These results suggest that consistent intake of BR extract might alleviate hypercholesterolemia and hepatic inflammation induced by excessive choline with a high-fat diet via lowering elevated levels of cecal TMA and serum TMAO in rats.

## 1. Introduction

Choline, one of the components of phospholipids in cell membrane and neurotransmitter, is regarded as an essential nutrient [1]. However, choline is also a precursor of trimethylamine-*N*-oxide (TMAO), which has been reported to act as a putative promoter of chronic diseases in human [2,3,4,5,6]. A part of excessive dietary choline is metabolized by gut microbiota to produce trimethylamine (TMA). Once TMA is absorbed from intestine, it is transported to liver via portal circulation and further oxidized to TMAO by hepatic flavin monooxygenases [2].

Since various epidemiological studies revealed connection between TMAO and cardiovascular diseases (CVD) [5,7,8], studies on TMAO and its precursors, such as choline, lecithin, and L-carnitine, have focused on vascular inflammation, endothelial dysfunction, and cholesterol homeostasis [3,4,5,9,10,11,12]. In addition, the effects of TMAO and its precursors on glucose intolerance [6] and hepatotoxicity [9,12] have been investigated. Taken together, it would likely be possible that TMAO can act in various organs throughout the body. More recently, TMAO has been demonstrated to induce expressions of cytokines and adhesion molecules in primary human aortic endothelial cells and vascular smooth muscle cells [3]. These inflammatory responses were also reported to be mediated via activation of nuclear factor-κB (NF-κB) signaling pathway, which is pivotal in inflammation, immunity, and cell death of various cell types [3].

Both epidemiological and experimental studies have revealed positive correlation between TMAO and chronic diseases such as CVD, renal disease, and diabetes [5,6,7,8,13,14,15,16]. Besides, evidences that TMAO might be able to cause hepatotoxicity or inflammation in adipose tissue have been provided [6,9,12]. However, consumption of fruits and vegetables has been widely known to be able to prevent incidence of chronic diseases. Phytochemicals, bioactive compounds in plants, contribute to reduce risks of those diseases mostly by their anti-oxidant activity [17].

Black raspberry (*Rubus occidentalis*; BR) is relatively high in anthocyanins among *Rubus* fruits [18]. It has been found that the major bioactive compounds in BR were anthocyanins, mainly cyanidin-3-rutinoside (C3R), cyanidin-3-glucoside (C3G), and cyanidin-3-xylosylrutinoside (C3XR) [19,20]. BR has been known to possess anti-oxidative, anti-inflammatory, and anti-cancer activities [21]. Especially, C3R and C3G were demonstrated to have anti-inflammatory activity through down-regulating NF-κB expression and inhibiting inhibitory κB (I-κB) degradation in lipopolysaccharide (LPS)-treated murine macrophages [19]. However, to the best of our knowledge, protective effects of polyphenols in BR on inflammation induced by excessive choline intake have not been reported.

The aims of this study were to investigate the effect of excessive choline intake on serum lipid profile and inflammation in rats fed high-fat diet and to evaluate the effect of polyphenols including anthocyanins in BR on choline-induced inflammation of the rats.

## 2. Materials and Methods

### 2.1. Materials and Chemicals

BR (*Rubus occidentalis*) fruits harvested in 2017 were purchased from Gochang, Korea. C3G, C3R, TMA, TMAO, and Folin-Ciocalteu reagent were the products of Sigma-Aldrich Chemical Co. (St. Louis, MO, USA). Choline chloride was obtained from Jinan Pengbo Biotechnology Co., Ltd. (Jinan, China). Trizol reagent was purchased from Invitrogen (Carlsbad, CA, USA). Radioimmune precipitation assay (RIPA) buffer and protease inhibitor cocktail (PIC) #6 were purchased from Biosesang Inc. (Seongnam, Korea). Anti-I-κB, anti-NF-κB, and horseradish peroxidase (HRP)-linked anti-rabbit immunoglobulin G (IgG) were purchased from Cell Signaling Technology (Danvers, MA, USA); anti-COX-2 from Novus Biologicals (Littleton, CO, USA); and anti-β-actin from Abcam (Cambridge, England). Enhanced chemiluminescence (ECL) solution was obtained from GenDEPOT (Katy, TX, USA).

### 2.2. Preparation of BR Extract

BR fruits (60 g) were crushed by hand and mixed with 80% (*v/v*) ethanol solution (300 mL) for 1 h by an overhead stirrer (WiseStir HS-30D, Daihan Scientific, Wonju, Korea). The extract was filtered with Whatman No. 2 filter paper (Whatman International Ltd., Maidstone, UK). The filtrate was concentrated using a vacuum rotary evaporator (A-10005, Eyela Co., Tokyo, Japan). The concentrate was freeze-dried using a freeze dryer (FDI06-85, Soritech, Hwaseong, Korea) to obtain powder form of the extract and stored at −20 °C for further studies.

### 2.3. Determination of Total Phenolic Content (TPC)

TPC in the BR extract was determined according to the method of Singleton et al. with a slight modification [22]. The BR extract (10 mg) was dissolved in 1 mL water followed by addition of 100 μL Folin-Ciocalteu reagent. After 3 min, 300 μL 20% (*w/v*) sodium bicarbonate solution was added to the mixture. The mixture was incubated at 40 °C for 30 min and then absorbance was measured at 765 nm by a spectrophotometer (Spectramax190, Molecular Devices, San Jose, CA, USA). TPC was presented as gallic acid equivalent (GAE). 

### 2.4. HPLC-UV Analysis of Anthocyanins in BR Extract

The BR extract powder (100 mg) was dissolved in 10 mL methanol containing 0.01% (*v/v*) hydrochloric acid. To separate anthocyanin fraction, 3 mL of the dissolved BR extract was injected into a Sep-Pak Plus C-18 cartridge (Waters Co., Milford, MA, USA), and the eluate was filtered using a 0.22 μm syringe filter (Pall Co., Port Washington, NY, USA). Composition and content of anthocyanins in the fraction were analyzed using a reversed-phase HPLC (Waters 2996 Separation Module, Waters Co., Milford, MA, USA) equipped with an XBridge C18 column (4.6 × 250 mm, 5 μm, Waters Co., Milford, MA, USA). Mobile phase was 5% (*v/v*) formic acid aqueous solution (A) and acetonitrile (B) with a gradient as follows: 0–1 min, 2% B; 1–2 min, 2–10% B; 2–15.5 min, 10–12.5% B; 15.5–21 min, 12.5–60% B; 21–26 min, 60–2% B; and 26–30 min, 2% B. Flow rate and column temperature were 1 mL min^−1^ and 30 °C. Anthocyanins were identified and quantified matching retention times of C3R and C3G standards at 520 nm. 

### 2.5. Animals and Diets

Forty female Sprague-Dawley (SD) rats (5 weeks old) were purchased from Koatech (Pyeongtaek, Korea). Female rats were selected since hepatic activity of flavin monoxygenase 3 is relatively higher in females than in males; therefore, they are prone to accumulation of TMAO in blood [5,23]. The rats were acclimatized to laboratory environment for 1 week under controlled temperature (23 ± 3 °C), humidity (50 ± 10%), and 12/12 h light-dark cycle. All the rats had free access to autoclaved tap water and normal chow diet during acclimation period of 1 week. After acclimated, they were randomly divided into 4 groups. Compositions of control AIN (American Institute of Nutrition)-93G diet and high-fat diet were shown in Table S1. High-fat diet supplemented with 0.6% BR extract was customized by Raonbio (Yongin, Korea). Treated groups were as follows: CON (AIN-93G diet (16% calories from fat)), HF (high-fat diet (45% calories from fat)), HFC (high-fat diet with 1.5% (*w/w*) choline water), and HFCB (high-fat diet with 1.5% (*w/w*) choline water and 0.6% BR extract). The CON and HF groups were given autoclaved tap water. All the animals were allowed free access to diet and water for 8 weeks and the water was replaced every two days. All protocols for animal experiment used in this study were conducted in accordance with institutional policies for animal health and well-being and approved by the Institutional Animal Care and Use Committee of Seoul National University (Approval No.: SNU-171103-1-5).

### 2.6. Blood and Tissue Collection

At the end of the experiment, all the rats were fasted for 6 h but allowed free access to water. All the animals were euthanized by asphyxiation with CO_2_. Blood was collected by cardiac puncture and centrifuged to get serum at 3000× *g* at 4 °C for 20 min after coagulation. Liver and adipose tissue were isolated and washed with saline. All the tissues were immediately stored at −80 °C until analysis.

### 2.7. Quantification of Choline, TMA, and TMAO

To determine the effect of choline intake on the production of choline-derived metabolites, cecal choline, TMA, and TMAO and serum TMAO were measured. To analyze the levels of choline-derived metabolites in cecum, cecal content was mixed with 80% (*v/v*) ice-cold methanol solution, vortexed for 5 min, and then centrifuged at 12,000× *g* for 5 min at 4 °C. The supernatant was filtered using a 0.22 μm syringe filter (Pall Co.) and the filtrate was concentrated by centrifugation (15,000× *g*, 25 min, 4 °C) in a Vivaspin centrifugal concentrator (Vivaspin 500, MWCO 3000, VS0192; Sartorius Stedim Lab, Stonehouse, UK). The concentrate was used for further analysis. Serum samples were mixed with 80% (*v/v*) ice-cold methanol solution, vortexed for 1 min, and then centrifuged at 12,000× *g* for 5 min at 4 °C. The supernatant was filtered using a 0.22 μm syringe filter (Pall Co.) and the filtrate was used for further analysis. 

All the analytes were separated on an Acquity UPLC (Waters Co., Milford, MA, USA) equipped with Acquity UPLC BEH amide column (2.1 mm × 100 mm, 1.7 μm, Waters Co., Milford, MA, USA) heated at 50 °C. Mobile phase consisted of two eluents: (A) 0.5 mM ammonium formate (pH 8.1) in water and (B) acetonitrile. The gradient program was: 0–2.5 min, 95–5% B; 2.5–5 min, 5–95% B; 5–6 min, 95% B. Flow rate was 0.6 mL min^−1^. The ion transitions (*m/z* 104.08 → 60.08 for choline; *m/z* 60.08 → 44.05 for TMA; and *m/z* 76.07 → 59.07 for TMAO) were used for quantitation. Samples were analyzed by SYNAPT G2-Si mass spectrometer (Waters Co., Milford, MA, USA) in positive ion electrospray mode. Capillary voltage and sampling cone voltage were set at 0.5 kV and 15 V, respectively. Flow rates of desolvation gas and cone gas were 650 L/h and 250 L/h, respectively. Desolvation temperature was 150 °C. Data acquisition and quantitation were carried out using MassLynx software 4.1 (Waters Co., Milford, MA, USA).

### 2.8. Serum Lipid Profile

Serum triglyceride (TG), total cholesterol (TC), and high-density lipoprotein-cholesterol (HDL-C) concentrations were determined with commercially available kits (Asan Pharmaceutical Co., Ltd., Seoul, Korea) according to the manufacturer’s instructions which are based on enzymatic colorimetric methods. Absorbance was measured by a spectrophotometer (Spectramax190, Molecular Devices). Serum low-density lipoprotein-cholesterol (LDL-C) level was calculated from Friedewald formula [24].

### 2.9. Total RNA Extraction, cDNA Synthesis, and Real-Time Quantitative Polymerase Chain Reaction (qPCR)

Total RNA were extracted from liver and adipose tissue using Trizol reagent according to the manufacturer’s instruction. Purity and quantity of RNA were evaluated by a NanoDrop spectrophotometer (NANO-200, Allsheng, Hangzhou, China). The RNA samples were reverse-transcribed using a GoScript Reverse Transcription kit (Promega, Madison, WI, USA) with random primers. qPCR was carried out with SYBR Green PCR Master mix (Applied Biosystems, Foster City, CA, USA) using Applied Biosystems StepOne Real-Time PCR system (Applied Biosystems, Foster City, CA, USA) under following conditions: 2 min at 95 °C for initiation, 15 s at 95 °C for denaturation, and 60 s at 60 °C for annealing up to 40 cycles. All qPCR primer sequences used in this study are listed in Table 1. All the relative expressions of genes were normalized to glyceraldehyde-3-phosphate dehydrogenase (GAPDH) expression and quantified using 2^−ΔΔCt^ method [25]. 

### 2.10. Western Blot Analysis

Liver tissue (100 mg) was homogenized with the mixture of RIPA buffer and PIC #6 at a ratio of 100:1 (1 mL) using a Tissuelyser (DE/85220, Qiazen, Hilden, Germany). The homogenized sample was agitated at 4 °C for 1 h and centrifuged at 12,000× *g* at 4 °C for 30 min (Smart R17, Hanil Scientific Inc., Gimpo, Korea). The supernatant was used to determine protein concentration using a modified Lowry protein assay kit (Thermo Fisher Scientific Inc., Waltham, MA, USA) according to the manufacturer’s instruction. The protein samples were loaded at 10 μL per well into 10% sodium dodecyl sulfate polyacrylamide gel and separated out at 60 V for 20 min and then at 120 V for 80 min. After the electrophoresis, proteins were transferred to nitrocellulose membrane (Bio-Rad Laboratories Inc., Hercules, CA, USA) at 370 mA for 100 min. Membranes were washed with Tris-buffered saline containing 0.1% (*v/v*) Tween 20 (TBST) and then blocked in blocking buffer (TBST containing 5% skim milk) for 1 h. 

Each of primary antibodies, anti-NF-κB, anti-COX-2, and anti-β-actin, was diluted to 1:500 in blocking buffer. Anti-I-κB was diluted to 1:250 in blocking buffer. Secondary antibody, HRP-linked anti-rabbit IgG, was diluted to 1:1000 in blocking buffer. After blocking, the membranes were incubated with primary antibodies on a shaker for 2 h. In turn, the membranes were washed 4 times for 5 min each using TBST and incubated with secondary antibody for 1 h. The membranes were then washed 4 times for 5 min each with TBST. Protein bands were visualized by ECL followed by densitometric analysis using Chemidoc XRS+ (Bio-Rad Laboratories Inc., Hercules, CA, USA).

### 2.11. Statistical Analysis

Results were expressed as means ± standard deviations. All statistical analyses were performed using SPSS program (version 23.0, SPSS, Chicago, IL, USA). Data were evaluated for normal distribution by means of Shapiro-Wilk test. Thereafter, either one-way analysis of variance (ANOVA) with Duncan’s multiple range test or Kruskal-Wallis test with Mann-Whitney U test was performed where applicable for analysis of differences among mean values at *p* < 0.05. 

## 3. Results and Discussion

### 3.1. Chemical Properties of the BR Extract 

A previous study reported that the major component of the BR extract using ethanol solution was carbohydrates (approximately 70% of the BR extract, wet basis) and small amounts of soluble proteins, ash, and anthocyanins [26]. Since biological properties of BR and its extract have been largely related to their phenolic-type phytochemicals [27,28], bioactive compounds in the BR extract used in this study would most likely be polyphenols.

In the present study, TPC in the BR extract was 42.7 ± 6.9 mg GAE g^−1^. Since C3XR standard was not commercially available, it was identified by comparison with chromatograms from Jung et al. [19] and presented as C3R equivalent (C3RE). The contents of C3XR, C3G, and C3R in the anthocyanin fraction were 0.83 ± 0.02 mg C3RE g^−1^, 0.50 ± 0.01 mg g^−1^, and 2.08 ± 0.08 mg g^−1^ (dry basis), respectively. C3R was the major anthocyanin accounting for 60% of total anthocyanins, which agrees with a previous study [19].

### 3.2. Body Weights and Food and Water Intakes

At the end of the experimental period, body weight and daily food intake were significantly higher in the CON and HF groups than in the HFC and HFCB (Table 2). This result might be due to the difference in food intake. Although notable difference was not observed between the body weights of the CON and HF, the HF group showed a significant increase in food efficiency ratio. In addition, no matter which became obese or not, hyperlipidemia, oxidative stress, and inflammation could be induced in high-fat diet-fed SD rats [29,30]. Meanwhile, supplementation of black raspberry resulted in a significant reduction in food efficiency ratio.

### 3.3. Serum TMAO Level and Cecal Choline, TMA, and TMAO Levels

**Figure 1:**
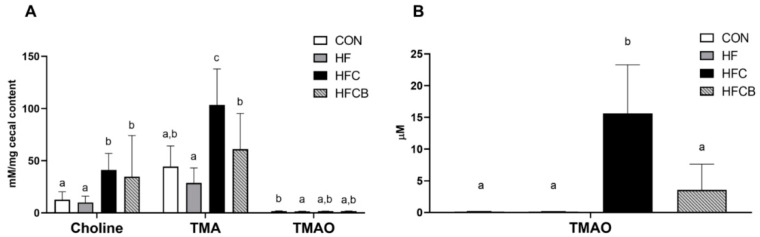
Effect of excessive choline intake on choline-derived metabolites in Sprague-Dawley rats. (**A**) Choline, trimethylamine (TMA), and trimethylamine-*N*-oxide (TMAO) in cecal content of the rats. (**B**) Serum TMAO level in the rats. All data represent the means and standard deviations (*n* = 8). Within the same metabolite, different small letters above bars indicate significant differences among the groups (*p* < 0.05; one-way ANOVA and Duncan’s multiple range test). CON (AIN-93G diet), HF (45% high-fat diet), HFC (HF + 1.5% choline water), and HFCB (HFC + 0.6% black raspberry extract).

The groups fed choline water showed significant higher level of choline in cecal content of the rats compared to the group fed autoclaved tap water (Figure 1A). The HFC group showed the highest level of TMA in cecum and supplementation of BR extract decreased the choline-induced elevated cecal TMA level. Likewise, serum TMAO level was significantly higher in the HFC group, while lower in the HFCB (Figure 1B). However, cecal TMAO levels were not significantly different when compared between the HFC and HFCB (Figure 1A). Dietary choline can be transformed into TMA by gut bacteria having TMA lyase (CutC) activity [31]. TMA produced once in the gut is absorbed from intestine and further oxidized to TMAO in liver. Therefore, it is necessary to reduce cecal TMA level for reduction of circulating plasma TMAO level. Some researchers reported that polyphenols, such as resveratrol, and probiotics, such as *Lactobacillus plantarum*, *Bifidobacterium animalis*, and *Enterobacter aerogenes* could be good sources to reduce elevated level of TMAO in blood by reduction of microbial TMA production [4,32,33,34]. There are also several studies showing that polyphenol-rich extract from various natural sources could have a prebiotic-like activity [35,36]. Therefore, the result suggests that BR extract rich in polyphenols, especially anthocyanins, might have potent to reduce cecal TMA level via modulation of gut bacteria.

### 3.4. Serum Lipid Profile

**Figure 2:**
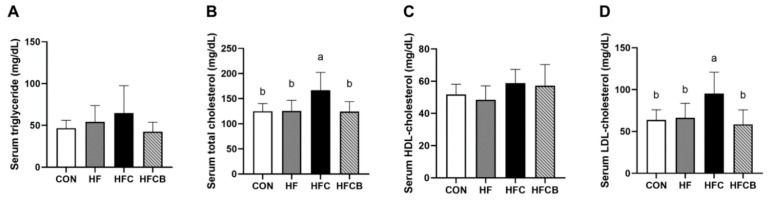
Serum triglycerides (**A**), total cholesterol (**B**), high-density lipoprotein (HDL)-cholesterol (**C**), and low-density lipoprotein (LDL)-cholesterol (**D**) in Sprague-Dawley rats. All data represent the means and standard deviations (*n* = 7–8). Different small letters above bars indicate significant differences among the groups (*p* < 0.05; one-way ANOVA and Duncan’s multiple range test). CON (AIN-93G diet), HF (45% high-fat diet), HFC (HF + 1.5% choline water), and HFCB (HFC + 0.6% black raspberry extract).

Serum TG level of the HFC group was 27.9% and 16.1% higher than those of the CON and HF, respectively, with no significant difference (Figure 2A). Serum TG level of the HFCB group was 21.6% and 34.3% lower than those of the HF and HFC, respectively, with no significant difference. It has been reported that intake of 3% choline water could elevate serum TG level in male Kunming mice [9,12]. However, in female LDL-receptor^−/−^ C57BL/6J mice, intake of 1.3% choline water did not change plasma TG level compared to control group [3]. It remains unclear whether and how excessive choline or TMAO intake affect blood TG level.

Serum levels of TC and LDL-C in the HFC group were higher than those in the CON, HF, and HFCB (*p* < 0.05), while these three groups had no significant difference (Figure 2B,D). There was no significant difference in serum HDL-C level among the groups (Figure 2C). The elevated serum TC level in the HFC group is in agreement with the results of Chen et al. [4] and Ren et al. [12], who reported that diet containing 1% choline and water containing 3% choline could raise serum TC in apolipoprotein E (ApoE)^−/−^ mice and healthy mice, respectively. It was suggested that the choline-induced elevation of serum TC might be because TMAO down-regulates expression of hepatic cholesterol 7 alpha-hydroxylase (CYP7A1), which is a key enzyme in bile acid synthesis from cholesterol [4].

In the present study, when the rats were fed both excessive choline and BR extract, serum TC level was significantly lower than the ones fed excessive choline alone. It was demonstrated that C3G intake could lower serum TC via up-regulating hepatic CYP7A1 expression in ApoE^−/−^ mice [37]. Therefore, BR extract rich in anthocyanins might lower choline-induced elevation of serum TC.

### 3.5. Relative mRNA Expression of Genes Involved in Inflammatory Response in the Liver and Adipose Tissue

**Figure 3:**
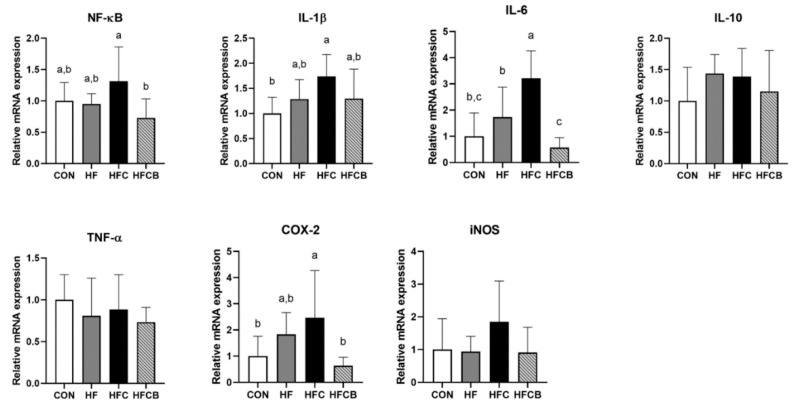
Relative mRNA level of genes involved in pro- and anti-inflammation in the liver of Sprague-Dawley rats. All the relative expressions of genes were normalized to glyceraldehyde-3-phosphate dehydrogenase expression. All data represent the means and standard deviations (*n* = 7–8). Different small letters above bars indicate significant differences among the groups (*p* < 0.05; one-way ANOVA and Duncan’s multiple range test). CON (AIN-93G diet), HF (45% high-fat diet), HFC (HF + 1.5% choline water), and HFCB (HFC + 0.6% black raspberry extract).

The mRNA expressions of NF-κB, interleukin (IL)-1β, IL-6, IL-10, tumor necrosis factor (TNF)-α, cyclooxygenase (COX)-2, and inducible nitric oxide synthase (iNOS) in liver and adipose tissue were determined by qPCR. NF-κB plays an important role in an inflammation response via regulating the expression of pro-inflammatory genes of cytokines, chemokines, and adhesion molecules [38]. In this study, the HFC group showed higher hepatic mRNA expression of NF-κB than the CON and HF (*p* > 0.05) (Figure 3). Although precise mechanism regarding effects of TMAO on NF-κB signaling pathway has not been clarified, trace amine-associated receptor (TAAR) 5, which is activated by TMA, has been suggested to be a possible mediator of TMAO activation due to the structural similarity between TMA and TMAO [3]. Also, another possible molecular mechanism has been suggested that uptake of TMAO into cells would mediate activation of NF-κB through collaborating with protein kinase C activator [11]. Despite these hypotheses, the exact mechanism of TMAO activity is still unclear. However, it might be able to regulate NF-κB expression in some ways. In the present study, NF-κB mRNA expression of the HFCB group was significantly lower than that of the HFC. Similar to this result, it was reported that anthocyanins from mulberry and sweet cherry (mainly C3G and C3R, respectively) down-regulate hepatic mRNA expression of NF-κB in diet-induced obese mice [39].

Once NF-κB is activated, it starts to induce inflammatory cytokines that can regulate immune response, such as IL-1β, IL-6, and TNF-α [38]. In the present study, hepatic mRNA expressions of IL-1β and IL-6 in the HF group were higher than in the CON (*p* > 0.05) and those in the HFC group were even higher than in the HF (*p* > 0.05) (Figure 3). Gao et al. [6] reported that mice fed high-fat diet containing 0.2% TMAO had higher mRNA expressions of those genes in epididymal adipose tissue than mice fed high-fat diet alone. In the present study, mRNA expressions of IL-1β and IL-6 in the HFCB group were markedly suppressed compared to the HFC. Likewise, intake of C3G-rich jaboticaba peel powder was able to suppress the expressions of IL-1β and IL-6 genes via decreasing phosphorylation of I-κB in liver of high-fat diet-fed mice [40]. IL-10 is known to be an anti-inflammatory cytokine inhibiting synthesis of pro-inflammatory cytokines such as IL-1, TNF-α, and interferon-γ secreted from macrophages and monocytes [41]. The mRNA expression level of IL-10 did not differ among all the groups in this study. In contrast to this result, intake of high-fat diet containing 0.2% TMAO decreased IL-10 mRNA expression in the epididymal adipose tissue of mice [6]. In the present study, there was no significant difference in mRNA level of TNF-α among all the groups. Effect of choline or TMAO intake on TNF-α mRNA expression has been reported to vary from organ to organ [3,6,10].

COX-2 and iNOS are highly inducible enzymes in specific circumstances associated with pro-oxidant and pro-inflammatory responses under regulation of NF-κB [42]. iNOS is regarded as a biomarker of inflammatory response because it can induce overexpression of nitric oxide, which can react with superoxide and further cause cytotoxicity [43]. In the present study, mRNA expression of COX-2 in the HFC group was higher than that in the HF (*p* > 0.05) and HFCB (*p* < 0.05) (Figure 3). However, there was no significant difference in mRNA expression of iNOS among all the groups. According to Seldin et al. [3], chronic intake of choline could up-regulate mRNA expression of COX-2 in aorta of atherosclerosis-prone LDLR^−/−^ mice.

**Figure 4:**
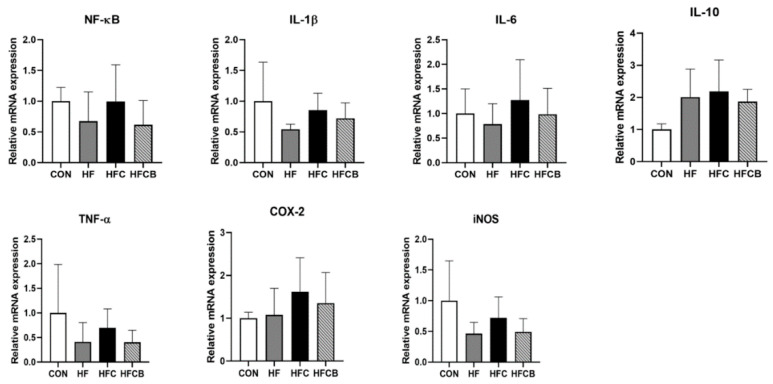
Relative mRNA levels involved in pro- and anti-inflammation in the adipose tissue of Sprague-Dawley rats. All the relative expressions of genes were normalized to glyceraldehyde-3-phosphate dehydrogenase expression. All data represent the means and standard deviations (*n* = 4–6). CON (AIN-93G diet), HF (45% high-fat diet), HFC (HF + 1.5% choline water), and HFCB (HFC + 0.6% black raspberry extract).

In the adipose tissue, mRNA expressions of IL-6 and COX-2 in the HFC group tended to be higher than in the CON and HF, and those in the HFCB group tended to be lower than those of the HFC (*p* > 0.05) (Figure 4). The mRNA levels of NF-κB, IL-1β, IL-10, TNF-α, and iNOS did not significantly differ among the groups. It was reported that the mRNA expressions of inflammatory cytokines such as IL-6 and IL-1β in epididymal adipose tissue were upregulated when the mice were fed high-fat diet containing 0.2% TMAO for 12 weeks [6]. Thus, previous studies have reported that inflammatory responses of macrophages in adipose tissue only occurred in prolonged (≥8 weeks) high-fat feeding in the rat [44,45,46]. Accordingly, long-term (≥8 weeks) experiment should be needed to evaluate the effect of excessive choline and BR extract on adipose tissue of rats fed high-fat diet.

Collectively, excessive dietary choline might exacerbate hepatic inflammation in rats fed high-fat diet via up-regulating mRNA expressions of NF-κB, IL-6, IL-1β, and COX-2. BR extract could ameliorate choline-induced inflammation via down-regulating those genes. However, eight weeks of experiment might not be enough to change the expressions of genes related to inflammatory response in adipose tissue of the rats fed high-fat diet with or without BR extract and choline.

### 3.6. Protein Expression of NF-κB, I-κB, and COX-2 in the Liver

**Figure 5:**
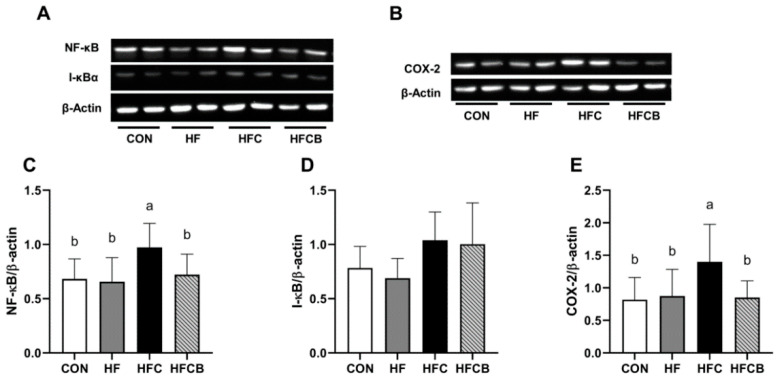
Protein expressions of NF-κB (**A**,**C**), I-κB (**A**,**D**), and COX-2 (**B**,**E**) in the liver of Sprague-Dawley rats. All data represent the means and standard deviations (*n* = 6–8). Different small letters above bars indicate significant differences among the groups (*p* < 0.05; one-way ANOVA and Duncan’s multiple range test). CON (AIN-93G diet), HF (45% high-fat diet), HFC (HF + 1.5% choline water), and HFCB (HFC + 0.6% black raspberry extract).

NF-κB dimer exists in the cytoplasm as an inactivated complex combined with I-κB. When cells are stimulated by specific stimuli such as antigen receptors, cytokines, reactive oxygen, and LPS, phosphorylation of I-κB occurs and then phosphorylated I-κB is degraded by protesome, releasing NF-κB dimer. NF-κB dimer then translocates into nucleus and binds to κB site of target genes [47]. In the present study, protein expression of NF-κB and COX-2 were significantly higher in the HFC group than in the CON, HF, and HFCB (Figure 5A–C,E). Similarly, it was reported that intake of C3R-rich black currant extract suppressed the hepatic protein expressions of NF-κB and COX-2 in diethylnitrosamine-initiated hepatocarcinogenesis of SD rats, as C3G-rich riceberry bran extract also did in gentamicin-induced liver damage [48,49]. Meanwhile, there was no effect of excessive choline or BR extract intake on the hepatic protein expression of I-κB (Figure 5A,D). In contrast to Jung et al. [19], who reported anthocyanins of BR could protect I-κB from LPS-induced degradation in macrophages, neither excessive choline nor BR extract affects protein expression of I-κB in this study.

## 4. Conclusions

Excessive choline can cause hypercholesterolemia and induce hepatic inflammation via, in part, NF-κB signaling pathway in rats fed high-fat diet. It might be due to elevated levels of cecal TMA and serum TMAO. Consistent intake of BR extract could lower the levels of cecal TMA and serum TMAO, which might result in the improvement of serum lipid profile in diet-induced hypercholesterolemia in rats. The result that BR could alter cecal TMA level suggests that BR polyphenols may act as a prebiotic in human gut as well. It could also alleviate hepatic inflammation via down-regulating the mRNA and protein expressions of genes related to inflammation. Further study, such as microbiome analysis, may be needed to elucidate the role of BR polyphenols, which seem to have a potent activity in reduction of cecal TMA level via modulation of gut bacteria.

## Figures and Tables

**Table 1:** Primer sequences used in real-time quantitative PCR.

Gene	Sequence
GAPDH	Forward (5′-3′): ACCACAGTCCATGCCATCAC
	Reverse (5′-3′): TCCACCACCCTGTTGCTGTA
NF-κB	Forward (5′-3′): TGGACGATCTGTTTCCCCTC
	Reverse (5′-3′): CCCTCGCACTTGTAACGGAA
TNF-α	Forward (5′-3′): GTAGCCCACGTCGTAGCAAAC
	Reverse (5′-3′): ACCACCAGTTGGTTGTCTTTGA
IL-6	Forward (5′-3′): TCCTACCCCAACTTCCAATGCTC
	Reverse (5′-3′): TTGGATGGTCTTGGTCCTTAGCC
IL-1β	Forward (5′-3′): GACTTCACCATGGAACCCGT
	Reverse (5′-3′): CAGGGAGGGAAACACACGTT
IL-10	Forward (5′-3′): GCTAACGGGAGCAACTCCTT
	Reverse (5′-3′): ATGTCCCCTATGGAAACAGCTT
COX-2	Forward (5′–3′): TGTATGCTACCATCTGGCTTCGG
	Reverse (5′-3′): GTTTGGAACAGTCGCTCGTCATC
iNOS	Forward (5′-3′): GCCATCCCGCTGCTCTAATA
	Reverse (5′-3′): GTTGGGAGTGGACGAAGGTA

GAPDH (glyceraldehyde-3-phosphate dehydrogenase), NF-κB (nuclear factor-κB), TNF-α (tumor necrosis factor-α), IL (interleukin), COX-2 (cyclooxygenase-2), and iNOS (inducible nitric oxide synthase).

**Table 2:** Body weights, weight gain, food and water intakes, and food efficiency ratio (FER) of rats.

	Group			
	CON	HF	HFC	HFCB
Initial body weight (g)	135.3 ± 8.9	137.1 ± 6.1	137.5 ± 7.5	137.0 ± 7.9
Final body weight (g)	249.2 ± 25.5 ^a^	255.5 ± 19.3 ^a^	214.0 ± 22.1 ^b^	207.6 ± 22.7 ^b^
Weight gain (g∙d^−1^)	2.0 ± 0.3 ^a^	2.1 ± 0.3 ^a^	1.4 ± 0.4 ^b^	1.3 ± 0.4 ^b^
Food intake (g∙d^−1^) *	26.5 ± 2.3 ^a^	24.7 ± 4.3 ^a^	18.3 ± 2.3 ^b^	17.6 ± 2.7 ^b^
Water intake (mL∙d^−1^) *	40.2 ± 4.3	41.7 ± 4.0	43.4 ± 1.9	39.3 ± 8.3
FER *	0.15 ± 0.01 ^a,b^	0.17 ± 0.01 ^a^	0.15 ± 0.01 ^a,b^	0.14 ± 0.02 ^b^

FER = weight gain (g∙d^−1^)/food intake (g∙d^−1^). Values represent means and standard deviations (*n* = 10). * *n* = 5. Values with different superscripts within each row are significantly different among the groups (*p* < 0.05; one-way ANOVA and Duncan’s multiple range test). CON (AIN-93G diet), HF (45% high-fat diet), HFC (HF + 1.5% choline water), and HFCB (HFC + 0.6% black raspberry extract).

## Supplementary Materials

The following are available online at https://www.mdpi.com/2072-6643/12/8/2448/s1, Table S1: Composition of experimental diets.

## References

[1] Zeisel S.H., Da Costa K.A. (2009). Choline: An essential nutrient for public health. Nutr. Rev..

[2] Zeisel S.H., Warrier M. (2017). Trimethylamine N-oxide, the microbiome, and heart and kidney disease. Annu. Rev. Nutr..

[3] Seldin M.M., Meng Y., Qi H., Zhu W., Wang Z., Hazen S.L., Lusis A.J., Shih D.M. (2016). Trimethylamine N-oxide promotes vascular inflammation through signaling of mitogen-activated protein kinase and nuclear factor-κB. J. Am. Heart Assoc..

[4] Chen M.L., Yi L., Zhang Y., Zhou X., Ran L., Yang J., Zhu J.D., Zhang Q.Y., Mi M.T. (2016). Resveratrol attenuates trimethylamine-N-oxide (TMAO)-induced atherosclerosis by regulating TMAO synthesis and bile acid metabolism via remodeling of the gut microbiota. MBio.

[5] Wang Z., Klipfell E., Bennett B.J., Koeth R., Levison B.S., Dugar B., Feldstein A.E., Britt E.B., Fu X., Chung Y.M. (2011). Gut flora metabolism of phosphatidylcholine promotes cardiovascular disease. Nature.

[6] Gao X., Xu J., Jiang C., Zhang Y., Xue Y., Li Z., Wang J., Xue C., Wang Y. (2015). Fish oil ameliorates trimethylamine N-oxide-exacerbated glucose intolerance in high-fat diet-fed mice. Food Funct..

[7] Wang Z., Tang W.H., Buffa J.A., Fu X., Britt E.B., Koeth R.A., Levison B.S., Fan Y., Wu Y., Hazen S.L. (2014). Prognostic value of choline and betaine depends on intestinal microbiota-generated metabolite trimethylamine-N-oxide. Eur. Heart J..

[8] Tang W.H., Wang Z., Levison B.S., Koeth R.A., Britt E.B., Fu X., Wu Y., Hazen S.L. (2013). Intestinal microbial metabolism of phosphatidylcholine and cardiovascular risk. N. Engl. J. Med..

[9] Jia M., Ren D., Nie Y., Yang X. (2017). Beneficial effects of apple peel polyphenols on vascular endothelial dysfunction and liver injury in high choline-fed mice. Food Funct..

[10] He Z., Lei L., Kwek E., Zhao Y., Liu J., Hao W., Zhu H., Liang N., Ma K.Y., Ho H.M. (2019). Ginger attenuates trimethylamine-N-oxide (TMAO)-exacerbated disturbance in cholesterol metabolism and vascular inflammation. J. Funct. Foods.

[11] Ma G., Pan B., Chen Y., Guo C., Zhao M., Zheng L., Chen B. (2017). Trimethylamine N-oxide in atherogenesis: Impairing endothelial self-repair capacity and enhancing monocyte adhesion. Biosci. Rep..

[12] Ren D., Liu Y., Zhao Y., Yang X. (2016). Hepatotoxicity and endothelial dysfunction induced by high choline diet and the protective effects of phloretin in mice. Food Chem. Toxicol..

[13] Organ C.L., Otsuka H., Bhushan S., Wang Z., Bradley J., Trivedi R., Polhemus D.J., Tang W.H., Wu Y., Hazen S.L. (2016). Choline diet and its gut microbe–derived metabolite, trimethylamine N-oxide, exacerbate pressure overload–induced heart failure. Circ. Heart Fail..

[14] Tang W.H., Wang Z., Kennedy D.J., Wu Y., Buffa J.A., Agatisa-Boyle B., Li X.S., Levison B.S., Hazen S.L. (2015). Gut microbiota-dependent trimethylamine N-oxide (TMAO) pathway contributes to both development of renal insufficiency and mortality risk in chronic kidney disease. Circ. Res..

[15] Missailidis C., Hällqvist J., Qureshi A.R., Barany P., Heimbürger O., Lindholm B., Stenvinkel P., Bergman P. (2016). Serum trimethylamine-N-oxide is strongly related to renal function and predicts outcome in chronic kidney disease. PLoS ONE.

[16] Koeth R.A., Wang Z., Levison B.S., Buffa J.A., Org E., Sheehy B.T., Britt E.B., Fu X., Wu Y., Li L. (2013). Intestinal microbiota metabolism of L-carnitine, a nutrient in red meat, promotes atherosclerosis. Nat. Med..

[17] Liu R.H. (2003). Health benefits of fruit and vegetables are from additive and synergistic combinations of phytochemicals. Am. J. Clin. Nutr..

[18] Torre L.C., Barritt B.H. (1977). Quantitative evaluation of *Rubus* fruit anthocyanin pigments. J. Food Sci..

[19] Jung H., Kwak H.K., Hwang K.T. (2014). Antioxidant and antiinflammatory activities of cyanidin-3-glucoside and cyanidin-3-rutinoside in hydrogen peroxide and lipopolysaccharide-treated RAW264.7 cells. Food Sci. Biotechnol..

[20] Paudel L., Wyzgoski F.J., Scheerens J.C., Chanon A.M., Reese R.N., Smiljanic D., Wesdemiotis C., Blakeslee J.J., Riedl K.M., Rinaldi P. (2013). Nonanthocyanin secondary metabolites of black raspberry (*Rubus occidentalis* L.) fruits: Identification by HPLC-DAD, NMR, HPLC-ESI-MS, and ESI-MS/MS analyses. J. Agric. Food Chem..

[21] Kula M., Krauze-Baranowska M. (2016). *Rubus occidentalis*: The black raspberry—Its potential in the prevention of cancer. Nutr. Cancer.

[22] Singleton V.L., Orthofer R., Lamuela-Raventós R.M. (1999). Analysis of total phenols and other oxidation substrates and antioxidants by means of folin-ciocalteu reagent. Meth. Enzymol..

[23] Bennett B.J., de Aguiar Vallim T.Q., Wang Z., Shih D.M., Meng Y., Gregory J., Allayee H., Lee R., Graham M., Crooke R. (2013). Trimethylamine-N-oxide, a metabolite associated with atherosclerosis, exhibits complex genetic and dietary regulation. Cell Metab..

[24] Friedewald W.T., Levy R.I., Fredrickson D.S. (1972). Estimation of the concentration of low-density lipoprotein cholesterol in plasma, without use of the preparative ultracentrifuge. Clin. Chem..

[25] Livak K.J., Schmittgen T.D. (2001). Analysis of relative gene expression data using real-time quantitative PCR and the 2−^ΔΔCT^ method. Methods.

[26] Shaddel R., Hesari J., Azadmard-Damirchi S., Hamishehkar H., Fathi-Achachlouei B., Huang Q. (2018). Double emulsion followed by complex coacervation as a promising method for protection of black raspberry anthocyanins. Food Hydrocoll..

[27] Jeong J.H., Jung H., Lee S.R., Lee H.J., Hwang K.T., Kim T.Y. (2010). Anti-oxidant, anti-proliferative and anti-inflammatory activities of the extracts from black raspberry fruits and wine. Food Chem..

[28] Seeram N.P. (2008). Berry fruits: Compositional elements, biochemical activities, and the impact of their intake on human health, performance, and disease. J. Agric. Food Chem..

[29] Wu Q., Li S., Li X., Sui Y., Yang Y., Dong L., Xie B., Sun Z. (2015). Inhibition of advanced glycation endproduct formation by lotus seedpod oligomeric procyanidins through RAGE–MAPK signaling and NF-κB activation in high-fat-diet rats. J. Agric. Food Chem..

[30] Xu Z.J., Fan J.G., Ding X.D., Qiao L., Wang G.L. (2010). Characterization of high-fat, diet-induced, non-alcoholic steatohepatitis with fibrosis in rats. Dig. Dis. Sci..

[31] Smaranda C., Emily P.B. (2012). Microbial conversion of choline to trimethylamine requires a glycyl radical enzyme. Proc. Natl. Acad. Sci. USA.

[32] Liang X., Zhang Z., Lv Y., Tong L., Liu T., Yi H., Zhou X., Yu Z., Tian X., Cui Q. (2020). Reduction of intestinal trimethylamine by probiotics ameliorated lipid metabolic disorders associated with atherosclerosis. Nutrition.

[33] Qiu L., Yang D., Tao X., Yu J., Xiong H., Wei H. (2017). *Enterobacter aerogenes* ZDY01 attenuates choline-induced trimethylamine *N-*oxide levels by remodeling gut microbiota in mice. J. Microbiol. Biotechnol..

[34] Qiu L., Tao X., Xiong H., Yu J., Wei H. (2018). *Lactobacillus plantarum* ZDY04 exhibits a strain-specific property of lowering TMAO via the modulation of gut microbiota in mice. Food Funct..

[35] Yang Q., Liang Q., Balakrishnan B., Belobrajdic D.P., Feng Q.-J., Zhang W. (2020). Role of Dietary Nutrients in the Modulation of Gut Microbiota: A Narrative Review. Nutrients.

[36] Loo Y.T., Howell K., Chan M., Zhang P., Ng K. (2020). Modulation of the human gut microbiota by phenolics and phenolic fiber-rich foods. Compr. Rev. Food Sci. Food Saf..

[37] Wang D., Xia M., Gao S., Li D., Zhang Y., Jin T., Ling W. (2012). Cyanidin-3-O-β-glucoside upregulates hepatic cholesterol 7α-hydroxylase expression and reduces hypercholesterolemia in mice. Mol. Nutr. Food Res..

[38] Tornatore L., Thotakura A.K., Bennett J., Moretti M., Franzoso G. (2012). The nuclear factor kappa B signaling pathway: Integrating metabolism with inflammation. Trends Cell Biol..

[39] Wu T., Yin J., Zhang G., Long H., Zheng X. (2016). Mulberry and cherry anthocyanin consumption prevents oxidative stress and inflammation in diet-induced obese mice. Mol. Nutr. Food Res..

[40] Dragano N.R., Cintra D.E., Solon C., Morari J., Leite-Legatti A.V., Velloso L.A., Maróstica-Júnior M.R. (2013). Freeze-dried jaboticaba peel powder improves insulin sensitivity in high-fat-fed mice. Br. J. Nutr..

[41] Hasko G., Szabó C., Németh Z.H., Kvetan V., Pastores S.M., Vizi E.S. (1996). Adenosine receptor agonists differentially regulate IL-10, TNF-alpha, and nitric oxide production in RAW 264.7 macrophages and in endotoxemic mice. J. Immunol..

[42] Liu D., Ji L., Wang Y., Zheng L. (2012). Cyclooxygenase-2 expression, prostacyclin production and endothelial protection of high-density lipoprotein. Cardiovasc. Haematol. Disord. Drug Targets.

[43] Aktan F. (2004). iNOS-mediated nitric oxide production and its regulation. Life Sci..

[44] Turner N., Kowalski G.M., Leslie S.J., Risis S., Yang C., Lee-Young R.S., Babb J.R., Meikle P.J., Lancaster G.I., Henstridge D.C. (2013). Distinct patterns of tissue-specific lipid accumulation during the induction of insulin resistance in mice by high-fat feeding. Diabetologia.

[45] Weisberg S.P., McCann D., Desai M., Rosenbaum M., Leibel R.L., Ferrante A.W. (2003). Obesity is associated with macrophage accumulation in adipose tissue. J. Clin. Investig..

[46] Xu H., Barnes G.T., Yang Q., Tan G., Yang D., Chou C.J., Sole J., Nichols A., Ross J.S., Tartaglia L.A. (2003). Chronic inflammation in fat plays a crucial role in the development of obesity-related insulin resistance. J. Clin. Investig..

[47] Luedde T., Schwabe R.F. (2011). NF-κB in the liver—linking injury, fibrosis and hepatocellular carcinoma. Nat. Rev. Gastroenterol. Hepatol..

[48] Bishayee A., Thoppil R.J., Mandal A., Darvesh A.S., Ohanyan V., Meszaros J.G., Háznagy-Radnai E., Hohmann J., Bhatia D. (2013). Black currant phytoconstituents exert chemoprevention of diethylnitrosamine-initiated hepatocarcinogenesis by suppression of the inflammatory response. Mol. Carcinog..

[49] Arjinajarn P., Chueakula N., Pongchaidecha A., Jaikumkao K., Chatsudthipong V., Mahatheeranont S., Norkaew O., Chattipakorn N., Lungkaphin A. (2017). Anthocyanin-rich riceberry bran extract attenuates gentamicin-induced hepatotoxicity by reducing oxidative stress, inflammation and apoptosis in rats. Biomed. Pharmacother..

