# Supplementary material for: Protective Effects of Black Raspberry (Rubus occidentalis) Extract against Hypercholesterolemia and Hepatic Inflammation in Rats Fed High-Fat and High-Choline Diets
Source: Nutrients. 2020 Aug 14;12(8):2448. doi: 10.3390/nu12082448 (PMC7468928; doi:10.3390/nu12082448)
Supplement: Supplementary file 1 [file nutrients-12-02448-s001.pdf]

Supplementary File

Table S1. Composition of experimental diets

Ingredient	Control diet		High-fat diet	
	Weight (g)	Energy (kcal)	Weight (g)	Energy (kcal)
Casein	200	800	233	932
L-Cystine	3	12	3.5	14
Corn starch	397	1590	84.8	339.2
Maltodextrin	132	528	116.5	466
Sucrose	100	400	201.4	805.6
Cellulose	50	0	58.3	0
Soybean oil	70	630	29.1	261.9
Lard	-	-	206.9	1862.1
TBHQ	0.014	0	-	-
Mineral mixture	35	0	11.7	0
Dicalcium phosphate	-	-	15.2	0
Calcium carbonate	-	-	6.4	0
Potassium citrate	-	-	19.2	0
Vitamin mixture	10	40	11.7	46.8
Choline bitartrate	2.5	0	2.3	0
Total	1000	4000	1000	4727.6
